# BOMS: blockchain-enabled organ matching system

**DOI:** 10.1038/s41598-024-66375-5

**Published:** 2024-07-11

**Authors:** Ikechi Saviour Igboanusi, Chigozie Athanasius Nnadiekwe, Joseph Uche Ogbede, Dong-Seong Kim, Artem Lensky

**Affiliations:** 1 ICT Convergence Research Center, Kumoh National Institute of Technology, Gumi, South Korea; 2 Department of IT Convergence Engineering, Kumoh National Institute of Technology, Gumi, South Korea; 3 Department of Biomedical Engineering, David Umahi Federal University of Health Sciences (DUFUHS) Uburu, Ohaozara, Ebonyi Nigeria; 4 Vascular Biology Program, Boston Children’s Hospital, Boston, MA USA; 5 Department of Surgery, Harvard Medical School, Boston, MA USA; 6 School of Engineering and Technology, The University of New South Wales, Canberra, ACT Australia; 7 School of Biomedical Engineering, Faculty of Engineering, The University of Sydney, Sydney, NSW Australia

**Keywords:** Blockchain, Cross-matching, Medical data, Organ matching, Smart contract, Biomedical engineering, Electrical and electronic engineering, Biochemistry

## Abstract

This work proposes a Blockchain-enabled Organ Matching System (BOMS) designed to manage the process of matching, storing, and sharing information. Biological factors are incorporated into matching and the cross-matching process is implemented into the smart contracts. Privacy is guaranteed by using patient-associated blockchain addresses, without transmitting or using patient personal records in the matching process. The matching algorithm implemented as a smart contract is verifiable by any party. Clinical records, process updates, and matching results are also stored on the blockchain, providing tamper-resistance of recipient’s records and the recipients’ waiting queue. The system also is capable of handling cases in which there is a donor without an immediate compatible recipient. The system is implemented on the Ethereum blockchain and several scenarios were tested. The performance of the proposed system is compared to other existing organ donation systems, and ours outperformed any existing organ matching system built on blockchain. BOMS is tested to ascertain its compatibility with public, private, and consortium blockchain networks, checks for security vulnerabilities and cross-matching efficiency. The implementation codes are available online.

## Introduction

For patients with advanced organ failure, organ transplantation is a critical and lifesaving medical treatment. However, demand for organ donations routinely outpaces supply, leading to long wait times, and avoidable and preventable deaths^1,2^. Interconnecting existing national donor systems into a single decentralized system would reduce the inefficiency of matching donors and recipients and aid in reducing deaths. The core of the organ donation system is an organ matching algorithm that plays a key role in increasing the rate of successful organ transplantations that takes into account a variety of factors, including tissue compatibility, immunological indicators, medical urgency, and geographical proximity^3^.

Organ-matching algorithms should preserve the data of all involved parties, as well as provide some level of tamper-resistant characteristics, especially when dealing with private medical information. To protect patient privacy and guarantee confidentiality, it is crucial to adhere to strict privacy standards^4^. The aforementioned requirements can be tackled by employing cryptographic techniques and distributed ledger technology.

Blockchain technology allows sensitive medical data to be shared and stored transparently and securely. In a recently proposed medical information system, the authors chose to store a cryptographic hash of the record on the blockchain to prevent tampering with the record^5^. The proposed system used smart contracts to automate and track changes in viewership rights or the creation of a new record in the system.

Smart contracts can be used in the context of organ matching to improve security and reliance on centralized servers. In particular, because the blockchain is decentralized and immutable, once the matching algorithm is employed and stored on it, it cannot be changed or tampered with^6,7^. By using an open-source smart contract, the transparency and immutability of the matching process are ensured. This promotes equity and trust among all parties involved.

Additionally, automated notifications and updates can be made available throughout the organ matching process thanks to smart contracts events-emitting property. For instance, when a suitable match is found, it can automatically send messages to the appropriate stakeholders, such as healthcare providers, transplant teams, and potential recipients.

While blockchain and smart contracts guarantee the algorithms are not being altered, the main downside is the need for the data being processed by the algorithm to be in plaintext. This makes it hard for privacy to be preserved, and hence donor matching should be performed on medical records that are visible to anyone. To reduce the chance of revealing the identities of patients, blockchain addresses are used to represent participants in the process. This provides some level of privacy while preserving transparency^8^.

This article focuses on the practical aspects of incorporating blockchain and smart contract technologies into organ donation systems. It considers regulatory issues, tackles implementation’s nuances, and examines biological processes involved in the matching process. The main contributions of this work include:

- implementation of a blockchain-enabled matching algorithm, and
- incorporation of several biological factors into the organ-matching process.

The rest of this paper is organised as below. “Literature narrative review” section presents the literature review. “Background” section is the background of blockchain technology and the biological considerations of organ donation. “System model” section presents the proposed system model highlighting the blockchain smart contract implementation design. The performance evaluation of the proposed system and its comparison to other existing systems are presented in “Evaluations and results” section. Finally, the conclusion and future works are presented in “Conclusion” section.

## Literature narrative review

Organizations such as the United Network for Organ Sharing (UNOS) and Eurotransplant (ET) are responsible for overseeing organ allocation and distribution in the United States and some countries in Europe, respectively. They collaborate with hospitals, transplant centres, and organ procurement organizations (OPOs) to regulate organ transplantation. UNOS utilizes a secured internet-based transplant information database system called “UNet” for organ allocation and transplant data management, including waitlists and donor lists. Both UNO and ET employ a point-scoring rule to assign points based on patient factors such as blood, waiting time, and distance from donors for organ allocation and donor management^9^.

Since 2014, the US kidney procurement organization has implemented the UNet allocation system to match donated grafts and recipients. This allocation system uses the kidney donor profile index (KDPI) and the estimated post-transplant survival score (EPTS) to optimize organ utility. The KDPI assigns numerical scores to the donor organ based on various factors, while the EPTS assesses the recipient’s likelihood of survival after transplantation based on specific recipient factors. Schulte et al. explored the applicability of these scores in the ET region and evaluated the effectiveness of the ET kidney allocation algorithm in promoting organ utility. Their findings indicated that only the donor age of the KDPI and the recipient age of the EPTS significantly predicted posttransplant graft and recipient survival^9^. However, the study concluded that the ET kidney allocation algorithm had limitations in matching graft and recipient due to poor age correlation.

The regulatory framework for organ donation and transplantation involves key entities such as the Uniform Anatomical Gift Act (UAGA) and the Organ Procurement and Transplantation Network (OPTN) in the United States^10,11^. The UAGA, established in 1968, serves as the legal basis for organ and tissue donations from individuals over 18 years of age^11^. Complementing this framework, it was noted that OPTN manages organ procurement and allocation through Organ Procurement Organizations (OPOs), which act as intermediaries between hospitals and UNOS associations during organ and tissue allocation^11,12^. In Italy, for example, the process of allocating deceased kidney donors begins at the regional level, where factors such as waiting time, age, HLA match, and percentage of panel reactive antibody (PRA) are considered before matching. This regional or national renal urgency system also takes into account combined transplant and pediatric priority before making allocations^13^.

In addition to government efforts, the Internet has become a significant source of organ donation. One prominent Internet-based organ allocation system that is gaining popularity is MatchingDonors.com^14^. Since its inception in 2004, MatchingDonors.com has facilitated thousands of transplants and currently boasts over 15,000 registered altruistic living donors as of 2023. The platform allows many patients to receive transplants within 6 months or less after registering on the website, with waiting times significantly shorter compared to donation networks managed by other organ procurement organizations^14^. Although the use of such platforms faced limited adoption initially due to unfamiliarity and perceived risks associated with the internet, MatchingDonors.com now receives over 1.5 million visits monthly. According to Henderson^15^, the Internet, especially social media, is also widely used for deceased organ donation and transplantation. Several solutions have been proposed to prevent illegal organ transplantation using a decentralized ledger^16,17^. Despite the broad application of various allocation systems, some countries still face challenges due to the lack of a connecting platform between donors, recipients, hospitals or procurement organizations. This gap could lead to illegal practices or illegitimate methods of allocation, including manipulation of waiting lists, tampering with patient data, and even trafficking.

Blockchain technology has significantly enhanced data sharing by ensuring data owners maximum security through encryption, immutability, and decentralization. Several, blockchain-based mobile applications have recently been developed for organ donation^6^ using a private Ethereum-based solution that enables organ donation and transplantation management that is decentralized, secure, traceable, auditable, private and trustworthy. In another web-based application, the matching was implemented on a first-in, first-out basis. However, patients in critical conditions skipped the queue^18^. Each patient had to go through an online registration process and provide medical ID, organ type, blood type as well as describe overall medical condition. In the review^19^, the authors highlighted the role of blockchain and distributed ledger technology in the field of organ transplantation. Waitlist manipulation was mitigated by employing decentralization technology.

## Background

### Smart contract

A smart contract is a programmable account in a blockchain network. A set of instructions are predefined in it before it is deployed. Once deployed, the instruction cannot be changed^20^. The smart contract in this system is equipped with functions that are used to register participants which include hospitals, donors, recipients, procurement organisations, doctors, transporters etc., organ progress tracking, donor/recipient matching, and the creation of the waiting list. After a successful match, the smart contract removes the matched donor and recipients from the list. The entire process after registration is managed by the smart contract. The system, when deployed on a public blockchain network, is highly secure. However, privacy is enhanced by using blockchain addresses to represent patients. The address is used to manage what each participant in the network can have access to. Hence operation can only be carried out by the authorized participants. The smart contract will serve as the backend of a decentralized application (DApp).

### Biological factors

On 3 September 2023, more than 103,000 potential organ recipients were on the US national transplant waiting list, and kidneys account for 86% of this number^21^. This creates a matching challenge and therefore requires a robust way to connect donors and recipients more efficiently. When matching donors and recipients for transplantation, several factors are considered and tests are performed to determine tissue and organ compatibility. The biological match determines the types of blood or antigens. The donor and recipient types of ABO blood need to be compatible for transplantation.

The human leukocyte antigen (HLA) matching is important to avoid immunological incompatibility and increase the chance of successful transplantation. Although HLA matching is largely required for kidney transplantation, heart donation based on HLA matching has been controversial, especially for deceased donors, due to the appearance of cold ischemia and clinical urgency^22^.

The size of the allograft is also considered in organ transplantation^23^, and a small organ size relative to the recipient has been found to result in reduced graft survival^24,25^. Since the size of the kidney is often not known at the time of offer, the height and weight of the donor and recipient, which are generally known, can be used to calculate the surface area of the body, and this is correlated with the size of the kidney^26^.

The age of the donor and recipient also plays a role in matching and graft survival. Although older recipients have shorter survival times even when a functioning organ is transplanted^27^, special considerations for transplantation are generally given to pediatric recipients compared to adults. To evaluate how age and graft size affect survival, the authors of^26^ proposed that age could attenuate the effect of donor-recipient size mismatch, and stated that the negative impact of donor-recipient size mismatch on graft survival was outweighed by the young age of the donor.

Another issue to consider in organ transplantation is the possibility of infection that might be transmitted to a recipient, as donor-derived infections are difficult to eliminate. Hence, examination of medical and social history as well as clinical testing such as viral load testing is usually adopted to minimize the transmission^28^.

Some other factors that are considered in organ matching include the geographical location of the donor with respect to the recipient’s location, how long potential recipients have been on the waiting list, and medical emergencies^29^. Special considerations are also given in cases where there is a previous living donor who later becomes a potential recipient.

### Factors affecting fair and transparent organ matching and allocation

There are several factors that must be considered to ensure fair and equal treatment of organ receivers.

1. *Utility*: Utility of all the benefits offered by the scarce resources. This approach involves the allocation of resources to benefit/save more lives (^30,31^). As we know, in an organ transplant there is always one organ, which implies one life to save. Therefore, factors such as who will benefit from the organ more will be considered and priority will be given based on
  - Patients who are likely to survive longest during the transplant process and after transplantation^30^.
  - Patients with the most compatibility ratio to avoid organ rejection^30^.
  - Availability of alternative treatment (post-clinical intervention)^31^
  - Graft or Organ survival period (how long the organ is likely to function after transplant)^32^.
2. *Equity*: In this case, the organs are allocated by random selection, which is done by lottery or first come first served.
  - Random selection, which means that the lucky recipient is determined through a lottery. This option seems to present a fair and unbiased selection process, but it can be disadvantageous to patients with more critical conditions as it may favour someone with the early-stage or fair condition or who may have longer years to be alive with the illness.
  - It also uses the first-come-first-serve rule, which will only favour those who have early knowledge and who were referred and registered early. Therefore, it does not consider the level of disease that makes it inappropriate and unfair to use in organ allocation and transplantation, as the decision is unfavourable to sicker patients.
3. *Allocation based on priority or preference for the most vulnerable patients*: This option considers patients with severe/worse conditions rather than relying on the waiting list^30^. Its disadvantage is that compatibility may not be factored in during selection, leading to the waste of scarce organs. This option assumes a temporary organ scarcity and presumes organ availability in the future for the less severe patients which is not guaranteed. It also gives priority to young patients (trying to prevent a short-lived life). These argue that more value would be given to the organ when allocated to a young patient than to an aged patient.
4. *Transparency and autonomy principle*: This option allows individuals to determine what they donate or receive as long as it does not impose harm on others^33^. With this, a patient can decide to refuse or accept a particular organ based on his or her belief or based on the donor’s health history. It also allows a donor or donor’s family to decide to donate directly if they are compatible, bypassing the allocation waiting list. The challenge with this option is that it has been exploited by patients who go about soliciting direct donation and even creating groups in which members promise their organs to others upon death, in return for other benefits which may include receiving the same if they need it.

## System model

### Proposed blockchain system model

**Figure 1:**
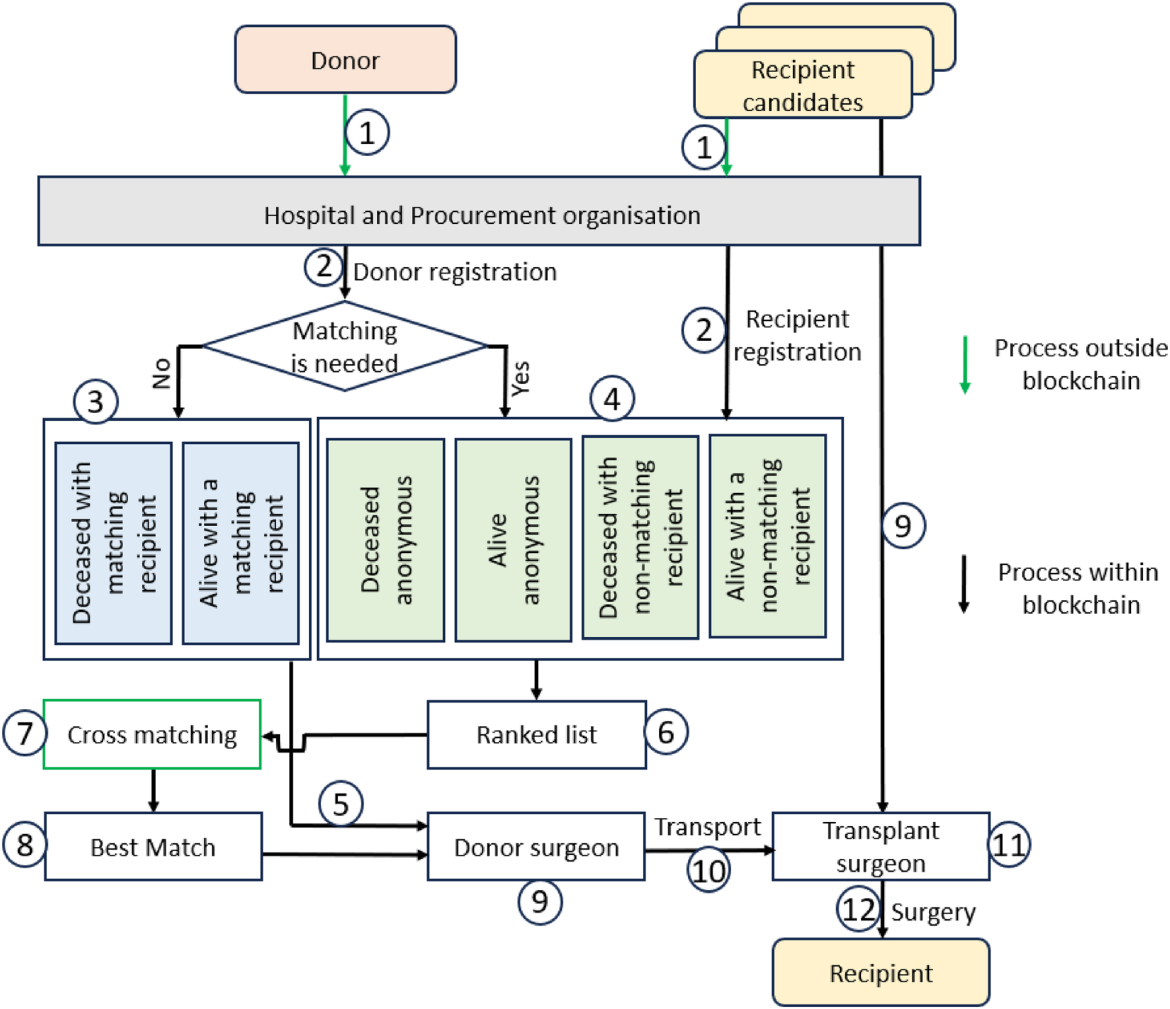
Entire proposed matching process depicting the registration process, the blockchain matching process and priority list generation, cross matching and surgery process.

The proposed organ matching system is built on blockchain using smart contracts. The biological factors are used to determine the compatibility of donors and recipients. Other factors such as location, fairness, and whether a recipient came with a donor are used to rank the recipients’ list. Figure 1 outlines the flowchart of the donation system. In every stage of the matching process, tracking is possible and is publicly accessible.

**Figure 2:**
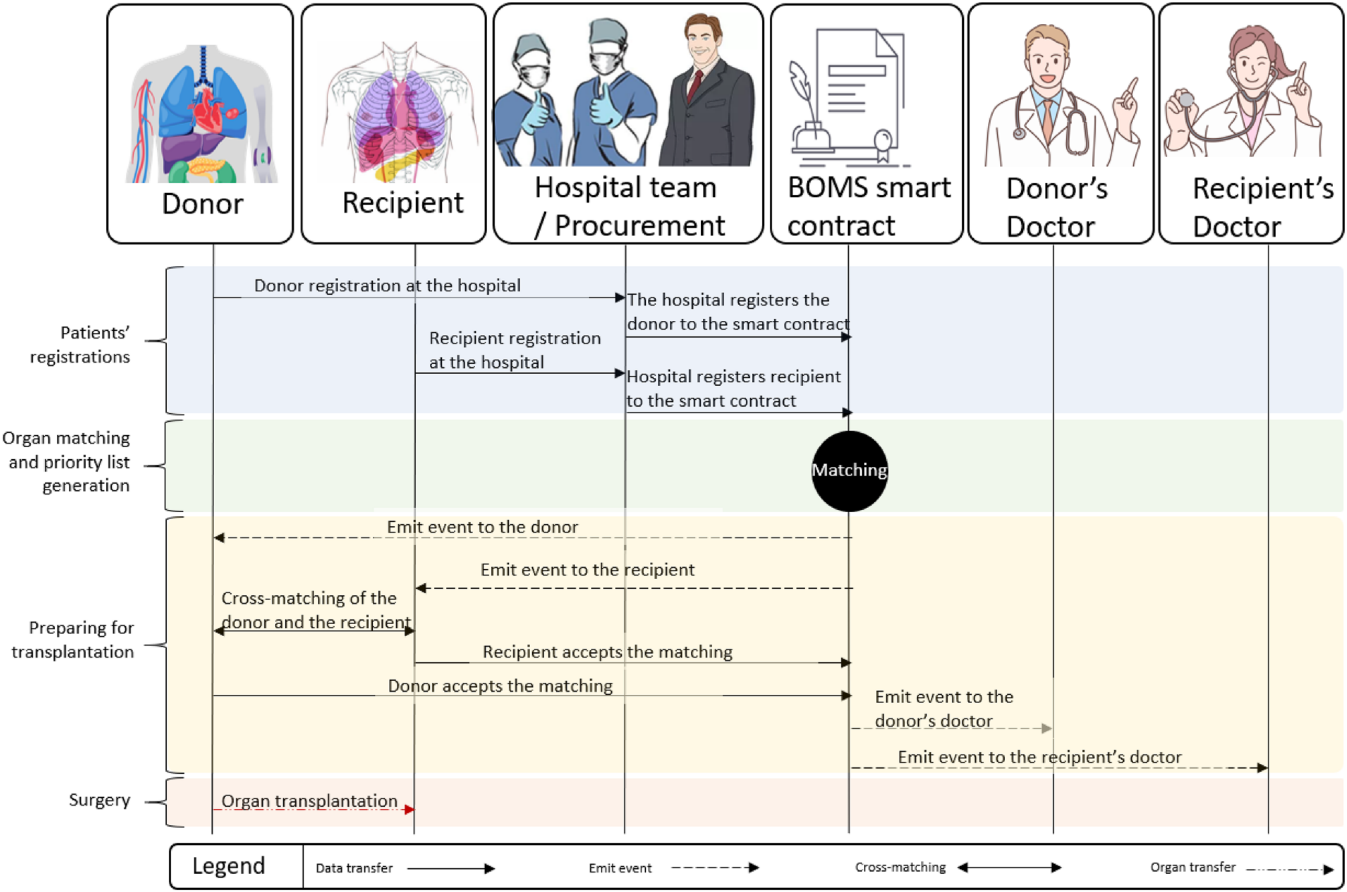
The diagram of the interaction between entities involved in the matching process, starting from the patients’ registration to the organ transplantation.

#### Registration by hospital

Both donors and recipients are registered through hospitals, and healthcare systems initiate the organ donation process^34^. The admitting hospital should comply with the following requirements:

- *Medical evaluation*: Hospitals have the competence and resources to conduct medical evaluations of potential donors and recipients. Donors must be tested to verify that their organs are transplantable, and recipients must be evaluated to confirm medical eligibility and compatibility with possible donors.
- *Legal and ethical principles*: Organ donation is governed by legal and ethical principles in several nations. Hospitals are frequently required by legislation to have processes in place to find potential donors, obtain consent, and ensure that the process is carried out ethically and legally.
- *Consent and assistance*: Hospitals can provide donors and recipients with proper counselling and assistance. This helps patients and their families make informed decisions about organ donation and transplantation while also considering the emotional and ethical components of the procedure.
- *Coordination*: Organ transplantation is a multistage process that includes donor identification, organ recovery, transportation, and transplant operation. Hospitals are well-equipped to organize these complicated logistics, ensuring that organ transplants are carried out in a timely and effective manner.
- *Public awareness*: Hospitals can help educate the public about the necessity of organ donation and transplantation. They can inform patients and their families about the advantages of registering as future donors or recipients, as well as resolve any misconceptions or concerns.
- *Standardization and documentation*: Hospitals can maintain consistent documentation of both donors and recipients, which is important for tracking and accountability in the organ donation process. This paperwork contributes to the transparency and fairness of the organ allocation procedure.
- *Accountability and oversight*: Hospitals are responsible for organ donation and transplant activities that take place within their facilities. Their participation ensures that the entire process is carried out with honesty, compliance with regulations, and oversight.

#### Organ donation process

To ensure that the transplanted organ is compatible with the recipient’s body i.e. reduces the risk of rejection and increases the likelihood of a successful transplant, the matching is performed by accounting for biological factors described in the Background section. The proposed system encourages recipients to come with a donor or vice versa, even if the pair is not compatible. Recipients and Donors can register without a pair; however, they will not get the points associated with pair registration. In the case of a living donor, authorisation is normally obtained from the donor, but in the case of a deceased donor, they give their consent before they are dead or their next of kin authorises before any organ or tissue donation can take place.

The two types of donors are distinguished in the proposed system:

i. *Donor that does not require matching:* As shown in Fig. 1, matching is not needed when the donor already has a predetermined compatible recipient. When the donor and recipient are already known to be compatible due to predetermined characteristics such as being closely biologically related or having passed extensive compatibility testing, matching is usually unnecessary. The compatibility between the donor and receiver is thoroughly checked before the surgery in many circumstances. While matching may not be necessary in situations where compatibility is well-established, the proposed system ensures the information related to such an operation is stored in the blockchain smart contract following the steps $\textcircled{1}$, $\textcircled{2}$, $\textcircled{3}$, $\textcircled{5}$, $\textcircled{9}$,$\textcircled{10}$, $\textcircled{11}$, and $\textcircled{12}$ shown in Fig. 1.
ii. *Donor that requires matching:* The proposed system has two major instances that require matching and they are:

- When a donor has a non-matching recipient: Matching is needed when the donor has a recipient but the recipient is not compatible. The donation of the organ is made successfully to the highest-ranked matched recipient. The recipient who is registered with the donor is set as a priority whenever a matchable donor is available. All information associated with this process is stored on the blockchain network. This situation will follow steps $\textcircled{1}$, $\textcircled{2}$, $\textcircled{4}$, $\textcircled{6}$, $\textcircled{7}$, $\textcircled{8}$, $\textcircled{9}$, $\textcircled{10}$, $\textcircled{11}$, and $\textcircled{12}$ shown in Fig. 1.
- *When a donor has no recipient (altruistic or nondirected donation):* Matching is needed when the donor has no predetermined recipient. Matching is done using extensive compatibility properties that ensure a high chance of success. The matching process prioritises recipients who initially registered with a non-matching donor. All information associated with this process is stored on the blockchain network. This situation will follow the same steps as “When a donor has a non-matching recipient” $\textcircled{1}$, $\textcircled{2}$, $\textcircled{4}$, $\textcircled{6}$, $\textcircled{7}$, $\textcircled{8}$, $\textcircled{9}$, $\textcircled{10}$, $\textcircled{11}$, and $\textcircled{12}$ shown in Fig. 1, however, no recipient receives the point for bringing the donor.

**Algorithm 1:**
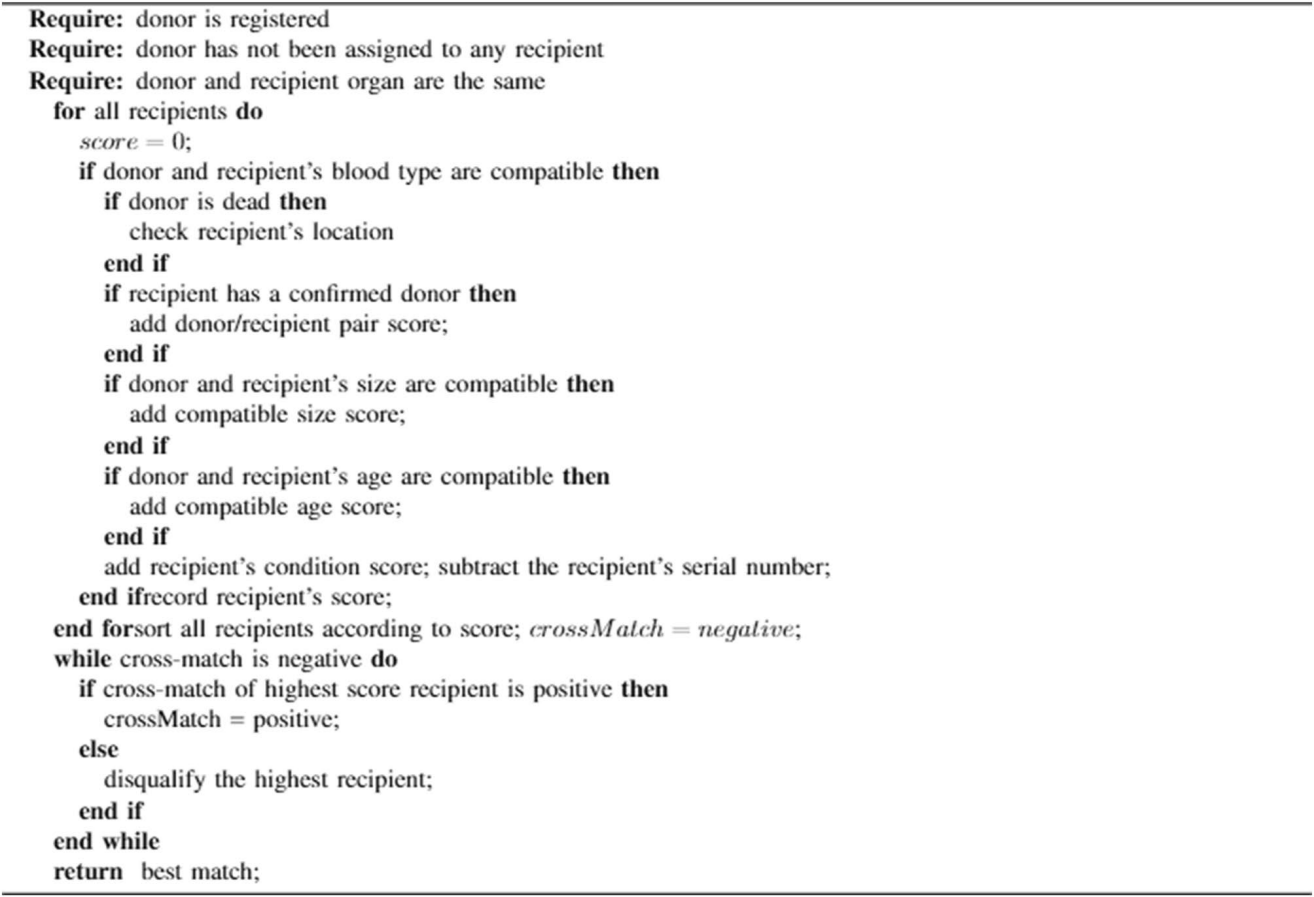
Donor recipient matching algorithm.

#### Donor and recipient matching process

The donor and the best matching recipient accept the matching before the donor’s and recipient’s surgeons are notified and the smart contract changes their state as matched. The interactions among entities in the proposed system are shown in Fig. 2.

All the processes from donors’ and recipients’ registration to the surgery are tracked using a blockchain smart contract.

### Biological model

**Figure 3:**
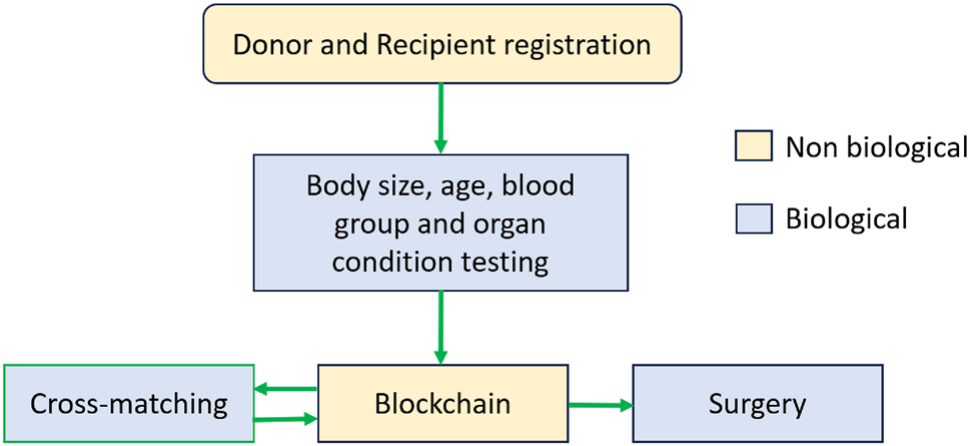
Flow of the biological process in the proposed BOMS.

Proper biological consideration increases the chance of successful organ transplantation. Figure 3 shows the flow of the biological and non-biological processes. To ensure a high chance of successful transplant our model has two stages of biological tests:

- *Pre blockchain matching* is the testing done before the initial data are sent to the blockchain matching system. This stage measures the size, age, organ condition and blood group of the patients. The blockchain model conducts the matching of patients using these pieces of information and other non-biological information to rank all the compatible patients to generate a ranked list.
- *Post blockchain matching*, a priority list is generated. From the highest priority recipient, a cross-matching test is conducted. The cross-matching process involves the examination of the blood samples of the donor and recipient under a controlled environment to ensure that the micro-interaction of cells can support organ transplantation.

To the best of our knowledge, this is the first work that considered pre and post-matching biological testing and integrating the processes with blockchain technology.

## Evaluations and results

The evaluations of this work focus on gas cost, security, comparison, user interface(UI), and the benefits and challenges of a blockchain-based organ donation system to non-blockchain-based ones.

**Table 1:** Smart contract and function gas cost.

Function	Operation	Gas
hospitalReqReg	Write	276,922
hospitalDonaReg	Write	255,413
matchListDonor	Write	528,978
matchListReci	Write	528,978
donorAcceptance	Write	48,435
recipientAcceptance	Write	56,331
track	Write	79,888
findDonorID	Read	11,129
findRecipientID	Read	14,550
viewDonor	Read	36,738
viewRecipient	Read	30,394
bestMatch	Read	12,891
viewTrack	Read	7854

**Figure 4:**
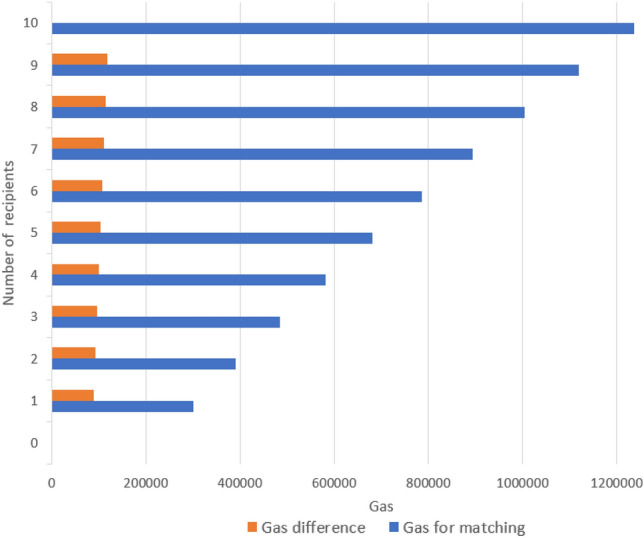
The analysis of the gas cost as the number of patients increases in the system. The blue bar represents (Gas for matching) the amount of gas the matching process requires when a certain number of recipients are on the smart contract waiting list. The orange bar is the additional gas required to match if a single recipient is added to the current waiting list.

**Figure 5:**
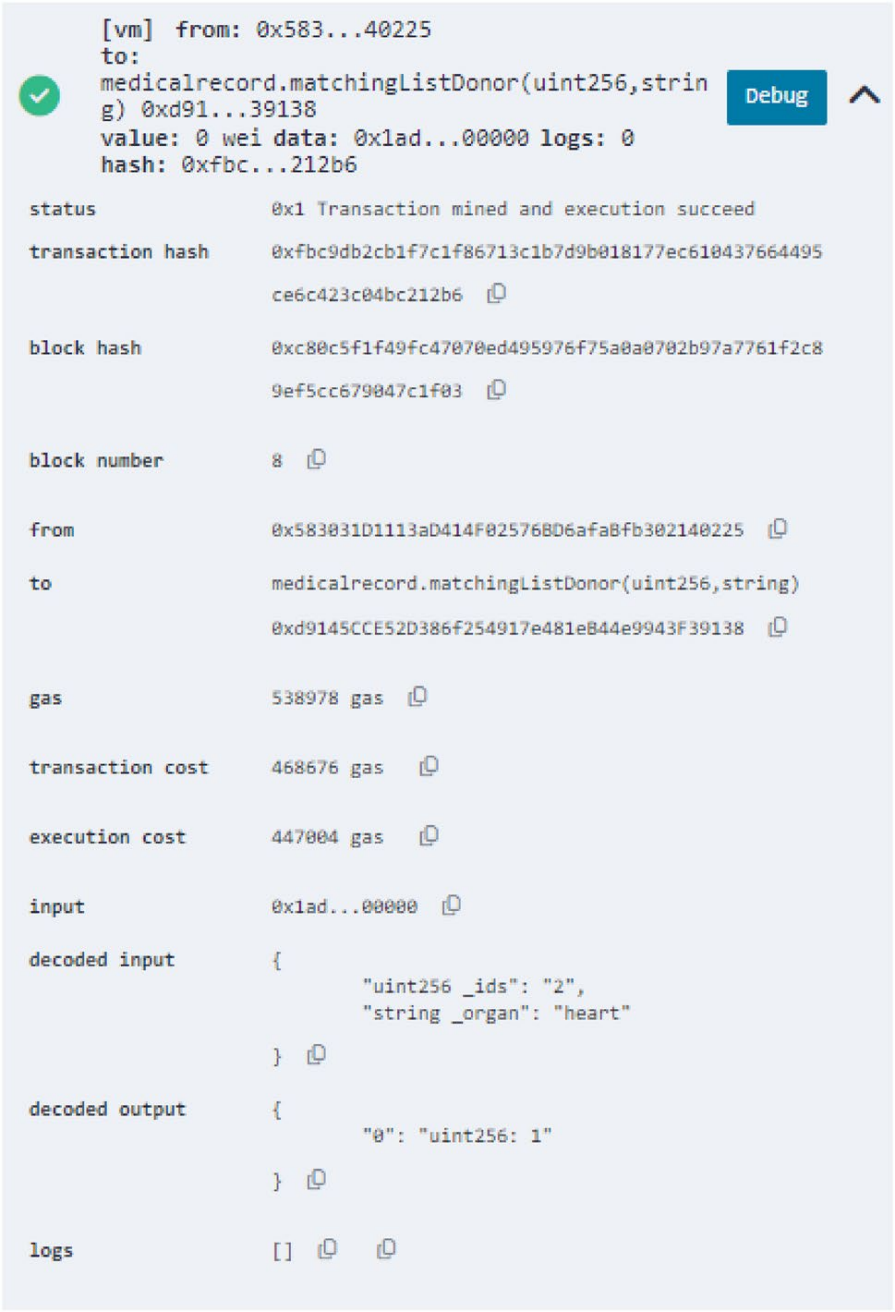
Log of a successful matching of a donor with a suitable recipient.

### Gas cost

There are fees associated with the computational activities carried out when smart contracts are deployed on the blockchain and when their functions are executed. We carried out a cost analysis concentrating on the gas consumption during the execution of the smart contract operations to evaluate the computational cost of our solution as shown in Table 1. In this investigation, we compared the gas expenses related to the various functions that we used to arrive at our solution. The cost of gas is a measure of the computational complexity of the functions, processes, and operations that are carried out by the Ethereum Virtual Machine (EVM). The write operation types consume more gas than the read operations because the write operations involve updating the blockchain record. The read gas cost is small compared to the write operations. The read operation cost is reduced to zero when a full node is reading the smart contract. Figure 4 presents the analysis of the gas cost as the number of patients increases in the system. The blue bar represents (Gas for matching) the amount of gas the matching process requires when a certain number of recipients are on the smart contract waiting list. The orange bar is the additional gas required to match if a single recipient is added to the current waiting list. When one patient is added, the gas cost increases in an identical pattern by an average of 104,180 gas. Figure 5 shows a successful matching operation.

### User interface

**Figure 6:**
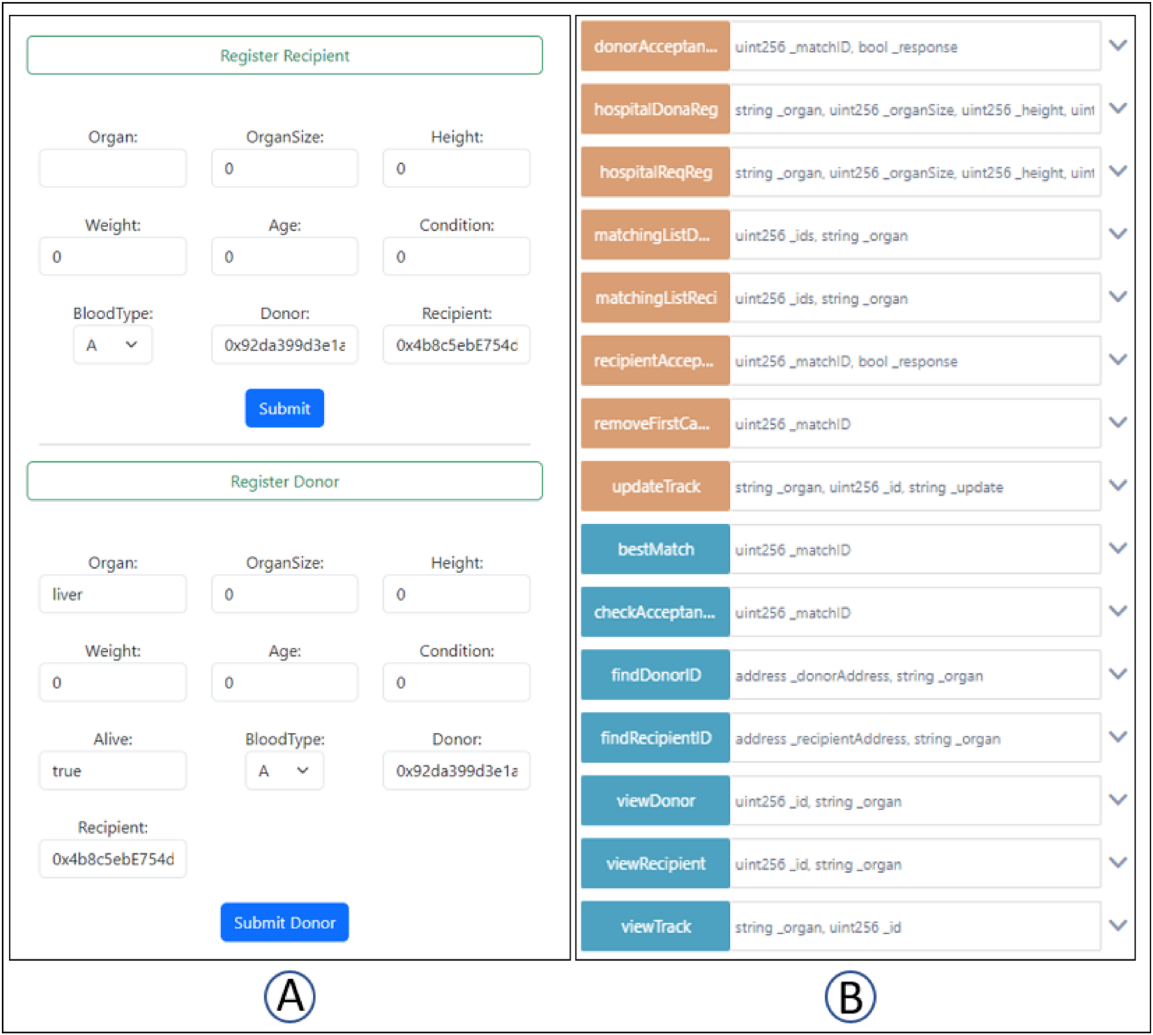
The proposed system can be interacted with through a customised user interface or an automatically generated user interface. (**A**) Is our customized design of the user interface for patient registration, and (**B**) is the user interface automatically generated by Remix IDE to test the smart contract.

A decentralized implementation of this work’s user interface (UI) facilitates communication between the blockchain smart contract and its user. It is the interactive interface of the web-based application. Figure 6A is the customised registration web page of our system, and Fig. 6B is the automatically generated Remix interface showing all the public functions of our system.

Some functions are not made public, hence they cannot be executed outside of the smart contract. These functions can only be called by other functions in the smart contract when certain conditions are met. These include the functions for sorting, blood group comparison, deactivation of successfully matched patients, and removal of recipients who refused to accept donor organ donation.

**Figure 7:**
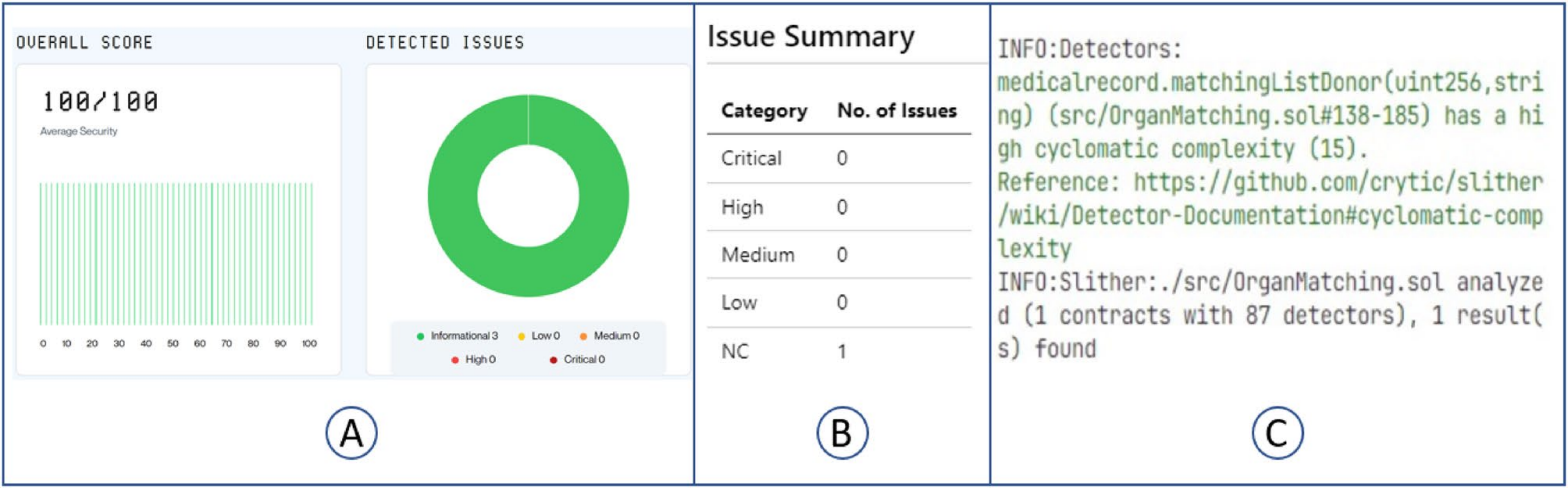
Smart contract vulnerability check results from three different platforms showing no vulnerability. (**A**) Is the check result using SolidCheck, (**B**) is the check result using Aderyn, and (**C**) is the check result using Slither.

**Table 2:** Comparison of the proposed system to other blockchain-enabled matching systems.

Features	Our solution	Hawashin et al.^6^	Alandjani^17^	Dajim et al.^18^.	Zouarhi^7^
Blockchain platform	Ethereum	Ethereum	NA	NA	Ethereum
Smart contract	Yes	Yes	Yes	No	No
Mode of operation	Public, private, and consortium	Private	Public	Public	Private
Tracing	Yes	Yes	Yes	Yes	Yes
Implementation	Yes	Yes	No	No	Yes
DApps	Yes	No	No	Yes	Yes
Cross-matching	Yes	No	No	No	No
Donors considered	Alive and dead	Alive and dead	Alive and dead	NA	NA
Security analysis	Yes	No	No	No	No
Non-matching donor and recipient	Considered	NA	Considered	NA	Considered

### Security

Blockchain technology inherently has the security provided by decentralization. However, some blockchain systems can have other forms of vulnerability. Smart contract implementation is a common source of security flaws. Our system has been examined with SolidCheck (https://solidcheck.io/), Aderyn (https://github.com/crytic/slither/), and Slither (https://github.com/Cyfrin/aderyn) smart contract vulnerability checkers, as shown in Fig. 7A–C respectively. The smart contract produced perfect security results, with a security score of 100%, when tested on SolidCheck. There are no issues detected by Aderyn vulnerability checker. Slither flagged a complexity warning, which is not a vulnerability, but a result of the complex process of comparing various properties of the patients to select the suitable candidates and rank them according to priority. This security evaluation attests to its suitability for secure data transfers throughout the intended blockchain ecosystem. The functions have access control features to ensure that only authorized users can execute certain functions. Some of the vulnerabilities checked by these smart contract vulnerability checkers include reentrancy attacks, front-running, integer overflow and underflow, simple logic errors, Block gas limit vulnerability, Default visibility, and timestamp dependencies. By eliminating these vulnerabilities the smart contract can be assumed to be secure. The testing of this system is performed on the Pure Chain Public EVM network, on a private Truffle Ethereum network, and on a Hyperledger Besu consortium network. The security check and the testing of our system on all three types of blockchain network operations suggest that our system is ready for use in diverse environments. These three Ethereum-based test environments are used because they represent typical public, private, and consortium blockchain environments.

### Comparison with other works

A comparison between the proposed system and some existing implemented blockchain-based solutions is shown in Table 2. The comparison is performed in terms of the platforms used, whether the security was achieved by means of smart contracts, the network operational principles, the capacity to trace the matching process, whether the implementation was present, whether DApps were implemented, whether cross-matching was considered, whether a possibility of having living and dead donors were accounted for, whether the security analysis was performed, and finally whether registration of non-matching donor and recipients was also considered. The proposed system is built on the Ethereum network and has been tested on public, private, and consortium modes of operation, while^6^ and^7^ were only implemented on the Ethereum private network and^17,18^ were deployed on public networks, but the details on exact networks were not provided. The proposed system as well as^6^, and^17^ are based on smart contracts.

Some systems presented front-end decentralized applications to interact with the matching process, while others did not^6,17^.

The proposed system as well as several others^6,17^ considered both living and dead donors, while others did not specify the target donor group. Our work is unique in performing a security check analysis and accounting for cross-matching.

The smart contract implementation code is available on GitHub and can be found in^35^.

### The benefits of blockchain-based organ donation systems

All the steps of the organ donation process are recorded on the blockchain. This allows the system to be:

- Transparent: Blockchain gives everyone involved in the organ transplant and donation process access to the same data and the ability to confirm its accuracy. By doing this, the likelihood of fraud, corruption, or manipulation of the organ allocation procedure can be eliminated^8^.
- Secure: Blockchain ensures that the data saved on the network is unchangeable and impenetrable through the use of cryptography and consensus techniques. This can protect the identities and privacy of donors and receivers and stop illegal access to or modification of the organ record^5–7^.
- Automation: Smart contracts are self-executing programs that can carry out predetermined actions based on specific situations. Blockchain makes this possible. In addition to enforcing the guidelines of the organ donation and transplantation system, this can automate the organ matching and delivery procedure^20^.

### The challenges of blockchain-based organ donation systems

The challenges and implications related to blockchain adoption in the organ-matching environment include:

- Scalability and performance: Performance and scalability are important technological barriers to blockchain adoption. Although it is possible to overcome these challenges by using layer two solutions, their implementation will increase the cost of setup and the complexity of the network^36^.
- Cost of Technology: Using a new technology necessitates initial investments for both the health institutions and patients, including learning expenses associated with becoming acquainted with the system. Also, the cost of buying the device that will serve as nodes in the blockchain network and the cost of energy to maintain the network is significant^37^.
- Ecosystem readiness: The ecosystem players lack awareness and understanding of blockchain’s benefits and usefulness. Adoption of blockchain will be difficult unless it is properly understood and accepted by decision-makers. The users both medical professionals and patients will require basic training to use the systems efficiently^37^.

## Conclusion

While the management of the organ donation system is a crucial process, that requires transparency, many organ donation systems are not transparent. To ensure transparency in the matching process, blockchain-enabled organ donation systems have been proposed.

Our proposed model handles the management of matching. By using patients’ electronic medical records, it selects the best match based on biological and geographical compatibility. This work to the best of our knowledge is the first work to have considered the role of cross-matching in the organ donation process and integrating it with a blockchain smart contract system.

Our system is designed to be practical, to ensure its usability in a real-world scenario. It has been implemented with a front-end decentralized application and has been successfully deployed and tested in public, private, and consortium networks. The smart contract implemented as part of the system has been tested using SolidCheck, Aderyn, and Slither security checkers and attained a 100 out of 100 security score, confirming that the smart contract is free from any known vulnerabilities.

In terms of the considered features, our model outperformed all of the previously proposed blockchain solutions while passing all the security checks.

In the future, to guarantee the privacy of donors’ and recipients’ medical records, we plan to employ homomorphic encryption. This would prevent unauthenticated use of patients’ medical records since currently all information on the blockchain network can be read by all members of the network. The scalability of the system can improve if there is a hybrid of cloud and blockchain networks. Resource-intensive computations are offloaded to the cloud, while activities that ensure security are managed by the blockchain network.

## Data Availability

The smart contract code generated and analysed during the current study is available in the GitHub repository, https://github.com/Ikechisaviour/BOMS-Blockchain-enabled-Organ-Matching-System. Any other data used or analysed during the current study are available from the corresponding author upon request.
